# Implementing the chronic care model for frail older adults in the Netherlands: study protocol of ACT (frail older adults: care in transition)

**DOI:** 10.1186/1471-2318-12-19

**Published:** 2012-04-30

**Authors:** Maaike E Muntinga, Emiel O Hoogendijk, Karen M van Leeuwen, Hein PJ van Hout, Jos WR Twisk, Henriette E van der Horst, Giel Nijpels, Aaltje PD Jansen

**Affiliations:** 1 Department of General Practice and Elderly Care Medicine/EMGO + Institute for Health and Care Research, VU University medical center, Amsterdam, the Netherlands; 2 Department of Epidemiology and Biostatistics/EMGO + Institute for Health and Care Research, VU University medical center, Amsterdam, the Netherlands

**Keywords:** Chronic care model, Frailty, Elderly, Primary care, Stepped wedge cluster randomised controlled clinical trial

## Abstract

**Background:**

Care for older adults is facing a number of challenges: health problems are not consistently identified at a timely stage, older adults report a lack of autonomy in their care process, and care systems are often confronted with the need for better coordination between health care professionals. We aim to address these challenges by introducing the geriatric care model, based on the chronic care model, and to evaluate its effects on the quality of life of community-dwelling frail older adults.

**Methods/design:**

In a 2-year stepped-wedge cluster randomised clinical trial with 6-monthly measurements, the chronic care model will be compared with usual care. The trial will be carried out among 35 primary care practices in two regions in the Netherlands. Per region, practices will be randomly allocated to four allocation arms designating the starting point of the intervention. *Participants*: 1200 community-dwelling older adults aged 65 or over and their primary informal caregivers. Primary care physicians will identify frail individuals based on a composite definition of frailty and a polypharmacy criterion. Final inclusion criterion: scoring 3 or more on a disability case-finding tool. *Intervention*: Every 6 months patients will receive a geriatric in-home assessment by a practice nurse, followed by a tailored care plan. Expert teams will manage and train practice nurses. Patients with complex care needs will be reviewed in interdisciplinary consultations. *Evaluation*: We will perform an effect evaluation, an economic evaluation, and a process evaluation. Primary outcome is quality of life as measured with the Short Form-12 questionnaire. Effect analyses will be based on the “intention-to-treat” principle, using multilevel regression analysis. Cost measurements will be administered continually during the study period. A cost-effectiveness analysis and cost-utility analysis will be conducted comparing mean total costs to functional status, care needs and QALYs. We will investigate the level of implementation, barriers and facilitators to successful implementation and the extent to which the intervention manages to achieve the transition necessary to overcome challenges in elderly care.

**Discussion:**

This is one of the first studies assessing the effectiveness, cost-effectiveness and implementation process of the chronic care model for frail community-dwelling older adults.

**Trial registration:**

The Netherlands National Trial Register NTR2160.

## Background

As a result of aging of the population of industrialized countries, the group of community-dwelling older adults with multiple chronic conditions is vastly expanding [1,2]. The subsequent accumulation of complex and long-term health needs causes the demand for care services to increase rapidly, accounting for a considerable share of health care utilization [3]. Globally, usual care is attempting to uphold the standards necessary to deliver high-quality chronic disease care. Pressure on the sustainability of health care systems is likely to further increase in the near future, demanding the identification and targeting of the main bottlenecks in care for frail older adults [4].

In Europe, three major barriers to high-quality care for frail community-dwelling older adults have repeatedly been identified over the past few years. First, our predominantly reactive care system fails to identify many older adults’ health risks and care needs at a timely stage, impeding the successful prevention of adverse outcomes. In addition, older adults experience a lack of autonomy in their own care process. Finally, care for frail older adults living at home is often fragmented, resulting in a lack of coordination and information exchange between health care professionals [5].

Offering integrated chronic care services may be the appropriate approach to overcome the challenges observed in elderly care: Evidence suggests that integrated care models have the potential to successfully improve quality of elderly care and may have a positive effect on health-related outcomes [6]. In addition, qualitative studies evaluating the environmental impact of such models report perceived benefits by informal caregivers [7]. However, overall review findings present a mixed picture, with studies showing inconsistent evaluation outcomes regarding the efficiency and effectiveness of the care models investigated [7-15].

The chronic care model is a multidimensional framework for chronic illness management, designed to guide and enhance the comprehensive and interdisciplinary delivery of care. Previous research has demonstrated its potential to improve health outcomes of patients with a chronic condition, and to improve quality of care [16-19]. Despite the fact that the chronic care model approach is widely used to implement integrated and long-term care services, to our knowledge only one study so far reported using the model to deliver care to community-dwelling frail older persons in a primary care setting [20].

To overcome the aforementioned barriers to high-quality care for older adults, the frail older Adults: Care in Transition (ACT) - study introduces the geriatric care model, a multifaceted intervention based on the chronic care model. Corresponding with the chronic care model, the geriatric care model aims to enable productive interactions between activated, informed patients and proactive, prepared health care professionals by combining in-home geriatric assessments with strong management by expert geriatric teams. To our knowledge, we are the only European study so far to investigate the impact of a chronic care model approach on frail older adults, and to evaluate the effectiveness as well as the cost-effectiveness and implementation process of such an intervention in a stepped wedge cluster randomised clinical trial.

Through implementation of the geriatric care model in a pragmatic trial, we seek to target the untimely recognition of health problems, the lack of autonomy perceived by older adults and the lack of coordination between health care professionals. In doing so, we hope to improve the quality of care for community-dwelling frail older adults, and subsequently improve their quality of life.

## Methods

### Study design and setting

**Figure 1:**
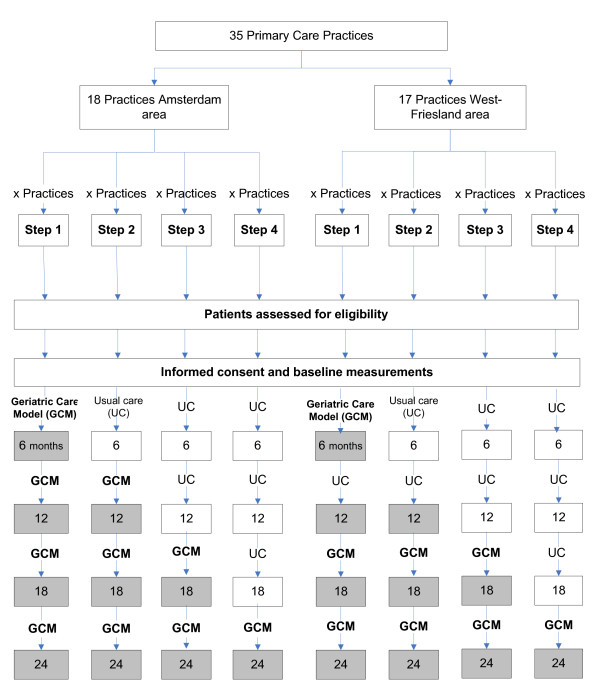
Flow diagram of the ACT-study.

We will implement the geriatric care model using a stepped-wedge cluster randomised clinical trial design. A stepped wedge design is a type of cluster randomized trial design involving sequential roll-out of an intervention to primary care practices (clusters) over a number of time periods [21]. Primary care practices in the control group offer usual care, whereas practices in the intervention group deliver care according to the geriatric care model. By the end of the study, all practices will have started with the intervention. The trial will run over a 24-month period, and will be conducted among a total of 35 primary care practices in the Netherlands, with practices distributed among the regions Amsterdam (18 practices) and West-Friesland (17 practices). The Amsterdam region is of an urban nature, whilst the West- Friesland area can be characterized as an urbanised rural setting. Following participant inclusion, effect measurements will be administered at baseline and at 6, 12, 18, and 24 months (Figure 1). The study received approval by the medical ethics committee of the VU University medical centre. Participants will enrol only after a signed declaration of informed consent.

### Randomisation

Per region, primary care practices will be randomised using the computer-based ‘Random Allocation Software’ program. In both regions, primary care practices will be allocated to one of a total of four allocation arms by randomization. The allocation arm number designates the starting moment of the intervention with the geriatric care model at practice level (at 0, 6, 12 and 18 months after baseline). Figure 1 shows the number of primary care practices per region, as well as the starting moment of the geriatric care model on cluster level.

### Study participants and recruitment methods

We will include community-dwelling frail older adults aged 65 years and over, recruited in participating primary care practices involved in the project, and their primary informal caregivers.

Frail older adults will be recruited in three steps.

1. Primary care physicians of participating primary care practices will identify frail individuals based on a composite definition of frailty (experiencing one or more limitations in either physical, psychological and/or social areas) in their population of patients age 65 years and over meeting a polypharmacy criterion: 5 or more drugs prescribed in the last 3 months [22]. Additionally, primary care physicians will include all other older patients meeting the composite description of frailty. Patients are excluded based on the following criteria: Residence outside area of practice registration; residence in a nursing home or in a home for the elderly; cognitive impairment or impaired mental status; critical or terminal illness.

2. Subsequently, all patients included by their primary care physician will receive an information letter and an informed consent form. Within two weeks, individuals selected during step one will be contacted by telephone by a project interviewer and asked to consider study participation.

3. In case of verbal consent, eligibility for trial entry will be established with the Program on Research for Integrating Services for the Maintenance of Autonomy case-finding tool for disability (PRISMA-7) [23]. Eligible patients (score ≥ 3) will be invited to participate in the study, whereupon an appointment will be made for administration of baseline measurements and the collection of the signed informed consent form by a project interviewer.

Primary informal caregivers will be recruited by inquiring with participating older adults. We define a primary informal caregiver as the caregiver carrying most of the care burden for the family member, relative or friend requiring care. If a primary informal caregiver is present, and the participating older adult does not oppose to their involvement in the study, eligible persons will be contacted by telephone and asked to enrol. Informal caregivers interested in participating will receive an information letter and an informed consent form.

### Intervention: geriatric care model

#### Rationale

**Figure 2:**
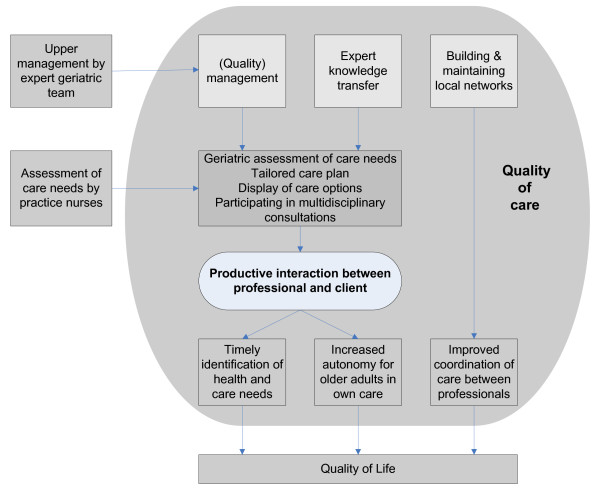
Schematic representation of the geriatric care model.

The geriatric care model aims to target three main challenges care for older adults is currently facing (i.e. untimely detection of older adults’ health risks and care needs, older adults’ lack of autonomy in their care process and inadequate coordination of care). We expect the geriatric care model to improve the quality of the organisation and delivery of care on structure, process and outcome levels. We expect the total impact of this process to improve patient outcomes, resulting in improved self-reported quality of life (Figure 2).

#### Geriatric care model

The geriatric care model aims to enable productive interactions between activated, informed patients and proactive, prepared health care professionals by combining tailored in-home geriatric assessments with strong management. In both the Amsterdam and West-Friesland region, the geriatric care model is integrated into routine practice by an expert geriatric team consisting of an experienced geriatric nurse and an elderly care physician.

#### Geriatric assessments by practice nurses

Every six months, a frail older adult will receive an assessment of health and care needs by a practice nurse, followed by a tailored care plan. This procedure will involve two home visits. During the first visit, a multidimensional assessment will be conducted using the web-based Community Health Assessment version 9.1 of the Resident Assessment Instrument (RAI-CHA) [24]. RAI facilitates the identification of existing care needs, helps nurses standardize their routines, and works as a reminder system for follow-up. After each RAI-assessment, the practice nurse will review the outcomes with the primary care physician and write a tailored care plan. Two weeks after the first visit a second home visit will take place, in which the nurse explores the older adult’s wishes regarding the outcomes of the assessment, provides them with information on appropriate management and/or treatment options, and stimulates their active involvement in the decision making process. According to their nature and content, actions listed in the final care plan will be evaluated by the older adult in consultation with the nurse. At all time, the older adult’s own care wishes will remain at the center of the decision making process.

#### Management by expert geriatric teams

During the intervention the geriatric expert teams will carry out the following three main tasks: (1) (quality) management, (2) expert knowledge transfer and (3) building and maintaining local networks of care organisations (Figure 2).

Management of the quality of care delivery by practice nurses and knowledge transfer will constitute of team meetings, training sessions and multidisciplinary patient reviews. First, team meetings will be held on a regular basis, and if required additional coaching and support will be provided to nurses individually. Additionally, as described in the paragraph below, geriatric team members will organize training sessions conform nurses’ educational needs. The sessions provide a platform for peer supervision and encourage knowledge exchange between nurses. Finally, in the event of a practice nurse reporting a complex patient, a multidisciplinary consultation will be organised by the geriatric team for an interdisciplinary review of the client’s situation. The consultation will be attended by a core group, consisting of the practice nurse, the primary care physician, the pharmacists and the geriatric team members. Depending on the situation demanding the consultation, other health care professionals central to the patient’s treatment (e.g. a physiotherapist) will be invited to join.

Throughout the intervention, the two geriatric teams will set up and maintain regional networks of local organisations. In order to facilitate the coordination between providers of care services for older adults in the region, primary care professionals and representatives of various community-based care organisations will meet on a regular basis, with the aim to subsequently enhance the coordination of care on a patient level.

#### Education of professionals

Practice nurses carrying out the intervention will take part in a tailored training program. Prior to the start of the intervention, nurses will participate in a 3-day motivational interviewing course. Further, both the practice nurses and the geriatric team members will participate in a one day RAI workshop before carrying out the intervention. In order to prevent contamination bias between the allocation arms, nurses will start their training program shortly before they start working with the geriatric care model. Alongside the intervention a second training program will run, consisting of a ‘training on the job’ motivational interviewing session as well as clinical education on geriatric topics provided by the expert team staff. Expert team members will identify each session’s topic(s) by means of field observations and RAI-output reviews. Several months into the intervention, practice nurses and the geriatric team members will be offered a refresher course RAI.

#### Strategies for implementation

Implementation of the geriatric care model is predicated upon the idea of the professional self-regulation of expert teams and practice nurses. Before the intervention is carried out its main theoretical and practical content is outlined by the research team, where after the development of work routines and protocols is informed by the professionals’ experiences in the field without research staff interference.

To avoid contamination, nurses working with the geriatric care model will not be employed in primary care practices in the control group that have yet to start delivering the intervention.

### Control group: usual care

Aimed at providing integral care services, primary care in the Netherlands plays an important role in the organisation of community elderly care. Of people over 75, almost hundred percent initiate contact with a primary care physician at least once a year [25]. Offering both (sub)acute and chronic care to older adults living at home, primary care physicians work in close collaboration with local health care services. A primary care physician may refer an older adult to a variety of local care organisations, whose services range from specialised in-home care to mental health support.

### Evaluating the geriatric care model

We will perform an effect evaluation, an economic evaluation, and a process evaluation. Throughout the 2-year data collection period older adults will advice researchers on the appropriateness and quality of the content of qualitative and quantitative research material, and will assist researchers with the interpretation of qualitative data derived from interviews with older adults.

### Effect evaluation

The primary outcome of the study is quality of life as measured by the 12-item Short Form questionnaire (SF-12) [26]. The SF-12 measures quality of life in two domains: a mental health component score (MCS) and a physical health component score (PCS). All outcome measures are presented in Table 1.

**Table 1:** Overview of outcomes, baseline measurements and follow-up measurements

**Older adults**		**Instrument**	**Baseline**	**6 Months**	**12 Months**	**18 Months**	**24 Months**
**Patient outcomes**	1. Quality of Life	SF-12 [26]	**x**	**x**	**x**	**x**	**x**
	2. Health-related Quality of Life	EuroQol (EQ-5D) [27]	**x**	**x**	**x**	**x**	**x**
	3. Independence in ADL	Katz ADL index [28]	**x**	**x**	**x**	**x**	**x**
	4. Psychological Wellbeing	RAND-36 subscale [29]	**x**	**x**	**x**	**x**	**x**
	5. Social Functioning	1 item on RAND-36 scale	**x**	**x**	**x**	**x**	**x**
	6. Self-reported Health	2 items on RAND-36 scale	**x**	**x**	**x**	**x**	**x**
	7. Acute Hospital Admissions	1 item of our data set and Cost Dairies	**x**	**x**	**x**	**x**	**x**
**Quality of Care**	8. Care Needs	CANE [30]	**x**		**x**		**x**
**Process Outcomes: Achieved transition**	9. Patient-reported Client-centred Care	CCCQ [31]	**x**	**x**	**x**	**x**	**x**
	10. Coordination of Care from the patient’s perspective	2 items on QUOTE [32]	**x**	**x**	**x**	**x**	**x**
**Informal Caregivers**		**Instrument**	**Baseline**	**6 Months**	**12 Months**	**18 Months**	**24 Months**
**Carer outcomes**	12. Quality of life	SF-12	**x**	**x**	**x**	**x**	**x**
	13. Self-rated Burden of Care	CarerQol [33]	**x**	**x**	**x**	**x**	**x**
**Older adults and informal caregivers**		**Instrument**	**Baseline**	**6 Months**	**12 Months**	**18 Months**	**24 Months**
**Costs**	Direct and Indirect Costs	Cost diaries					

*SF-12* Short Form-12 questionnaire; *EuroQol (EQ-5D)* measures of 5 dimensions of health-related quality of life; *Katz ADL* Katz Activities of Daily Living; *RAND-36* measure of health-related quality of life; *CANE* Camberwell assessment of needs in the elderly; *Arm 1-4* allocation arm number 1–4; *CCCQ* Client-centred Care Questionnaire; *QUOTE* QUality Of care Through the patient’s Eyes; *CarerQol* care-related quality of life of informal caregivers

#### Sample size calculations

Sample size calculations are based on the expected effects of the intervention on the primary outcome, quality of life, measured by the SF-12. Calculations are done using the equation for a longitudinal design [34] and adjusted for the effect of clustering of primary care practices [35]; SF-12 coefficients used in the calculations are derived from previous studies with a study population similar to ours. The number of follow up measurements is 4; Mean cluster size is based on the expected number of participants per cluster at baseline (N = 33). We assume the following: a standard deviation of 7.1 (PCS and 6.6 (MCS) [36], a correlation coefficient of repeated measurements of 0.66 (PCS) and 0.5 (MCS) [37], an intra-cluster correlation coefficient of 0.02 for both PCS and MCS [36], an alpha of 0.05 and a power of 90%. To detect a clinical difference of 3 points [29] on the SF-12 scale between intervention and control groups and assuming an attrition rate of 20%, the study would require 180 (PCS) and 131 (MCS) eligible patients per arm.

#### Data collection

Data will be collected among older adults at home by means of computer assisted personal interviewing. For each observation, interviewers will receive training and will be supervised by research staff. A random sample of the interviews will be tape recorded; In order to enhance the quality of the data, researchers will evaluate the quality of the recordings and report back their findings to the interviewers. We will use postal questionnaires to collect data among the primary informal caregivers.

#### Analyses

First, characteristics of the participants at baseline will be described and differences between the 4 allocation arms are tested using chi-square tests and ANOVA or non-parametric tests.

Second, effect analyses will be done based on the “intention-to-treat” principle. For both older adults and their primary caregivers, all outcome measures will be compared between the group receiving the geriatric care model and the group receiving usual care using multilevel regression analysis. Multilevel regression analysis takes into account the non-independent nature of hierarchical data and does not require data for a fixed number of observations for all respondents [38]. In the present study, three levels can be distinguished. Repeated observations are clustered within the patient and the patients are clustered within the primary care practices. Potential confounding (due to baseline differences) and effect-modifying will be accounted for during the analysis. If necessary an adjustment will be made for baseline differences between the groups.

In addition to the investigation of the overall effect of the intervention, the stepped-wedge design allows us to study the effects of the duration of the intervention on outcome measures [39]. Therefore, in additional analysis, the interaction between time and intervention will be added to the analyses.

#### Blinding

We will aim to maintain a blind status for as many people involved in the study as possible. Professionals carrying out the intervention will not be informed about a patient’s enrolment in the study until the start of the intervention. During data analysis, researchers will be blinded to the group assignment. Due to ethical considerations, it will not be feasible for interviewers collecting the data and participants to be blinded to group assignment. All participants will be informed about the starting time of the intervention, and will at all times be aware of their group status.

### Economic evaluation

The economic evaluation will be performed from a societal perspective. We will consider all relevant direct and indirect costs, such as costs of the care model, consultations with primary care physicians, medical specialists, home care, medication use, hospital and nursing home admissions, informal care time and cost of lost labour days of the informal caregiver. Care utilization data of both the client and the caregiver will be prospectively collected alongside the trial using six monthly cost diaries. Medication data of clients will be obtained from the centralized pharmacy files in the research regions. Dutch standard costs are used to value resource use [40,41]. Medication costs will be valued using prices of the Royal Dutch Society for Pharmacy [42]. Lost productivity costs will be calculated according to the friction cost approach (friction period 154 days) using the mean age and sex specific income of the Dutch population [40,43]. We will calculate a cost price for the care model using a bottom-up approach. The EuroQol (EQ-5day) [27] will be used to measure health-related quality of life. We will estimate utilities with the Dutch tariff developed by Lamers et al. [44] and then calculate Quality Adjusted Life Years (QALYs).

Missing data on costs and outcomes will be imputed using multiple imputation according to the Multivariate Imputation by Chained Equations (MICE) algorithm [45]. A cost-effectiveness (CE) analysis will be performed comparing the difference in total mean costs to the difference in quality of life, functional status and care needs; a cost-utility (CU) analysis will be used to estimate the incremental costs per QALY. Uncertainty around the incremental CE and CU ratios will be estimated using the bias-corrected percentile bootstrapping method (5000 replications) and will be plotted in cost-effectiveness planes. In addition, cost-effectiveness acceptability curves and net monetary benefits will be estimated to show the probability that the geriatric care model is cost-effective in comparison with usual care using different ceiling ratios [46]. Sensitivity analysis will be done to assess the robustness of the results and will include the most important cost drivers.

### Process evaluation

Alongside the intervention, we will conduct a mixed methods process evaluation. The process evaluation data will be used for summative purposes: Information will be used to investigate the extent to which the intervention was implemented as planned [47]. By exploring the ‘black box’ of the intervention, we aim to both gain an insight in (cost)effectiveness results and facilitate future implementation.

Process outcomes are level of implementation of the geriatric care model, barriers and facilitators to successful implementation, and the extent to which the intervention manages to achieve the transition necessary to target the three challenges in care for older adults, i.e. the untimely identification of health problems and care needs, the lack of client autonomy and the inadequate coordination between care professionals.

First, we will aim to assess the level of implementation using process constructs fidelity, dose delivered and dose received [47]. Further, we will seek to identify barriers and facilitators to successful implementation of the geriatric care model on cultural, operational and structural levels. To investigate whether the geriatric care model accomplishes the transition we intended, we will explore how older adults experience autonomy, how older adults and health care professionals experience the way care is coordinated during the intervention, and the extend to which the geriatric care model manages to be proactive in indentifying previously undetected health problems and care needs. Finally, in order to facilitate future implementation of the geriatric care model, we will investigate health care professionals’ learning experiences with the intervention. Table 2 offers an overview of process outcomes, used constructs and methods of data collection.

**Table 2:** Process outcomes, constructs and methods of data collection

**Outcome**	**Concept**	**Methods**
**Level of implementation**	Fidelity	·Semi-structured interviews* with geriatric team members, nurses, primary care physicians ·Focus groups* with practice nurses · *Tailored care plans, time registration by practice nurses, registrations by expert geriatric teams, minutes of team meetings and training sessions*
	Dose delivered (completeness)	·*Tailored care plans* Total number of care plans delivered to clients during the intervention · *Tailored care plans (sample)* Average number of observations (RAI outcomes, nurses’ own observations) per care plan Average number of actions formulated per care plan Average number of care professionals involved in actions listed ·*Time registrations by practice nurses* Average amount of nurse’s working hours spent delivering intervention components (e.g. In-home assessments and care plan evaluation with clients, consultations with primary care physician, multidisciplinary consultations) ·*Registrations by expert geriatric teams* Number of multidisciplinary consultations organized
	Dose received (exposure, satisfaction)	·*Minutes of team meetings, registrations by expert geriatric teams* Number of training and coaching sessions and frequency of nurse’s attendance at these sessions Number of team meetings and frequency of nurse’s attendance at these meetings ·Semi-structured interviews* with geriatric team members, nurses, primary care physicians ·Focus groups* with practice nurses, local stakeholders
**Barriers and facilitators to implementation**	Barriers on cultural, operational and structural levels	·Semi-structured interviews* with older adults, geriatric team members, nurses, primary care physicians ·Focus groups * with practice nurses, stakeholders
**Extent to which transition is achieved**	Client autonomy	·Semi-structured interviews * with older adults ·CCCQ questionnaire **
	Coordination of care from the perspective of health professionals and patients	·Semi-structured interviews * with geriatric team members, nurses, primary care physicians, older adults ·Focus groups * with practice nurses ·2 Items on QUOTE questionnaire **
**Learning experiences of professionals**	Timely identification of health problems and care needs	·*Tailored care plans* Percentage of total number of RAI outcomes and nurses’ own registrations previously unknown to heath care professionals
		·Semi-structured interviews * with geriatric team members, nurses, primary care physicians · Focus groups * with practice nurses, stakeholders

* The qualitative study sample will include all older adults and health care professionals participating in the project, i.e. practice nurses, geriatric team members and primary care physicians and will be selected by means of a purposive (maximum variation) sampling procedure [48]; ** The quantitative study sample will include all older adults participating in the study; *CCCQ* Client-centred Care Questionnaire; *QUOTE* QUality Of care Through the patient’s Eyes.

Per process outcome, results of the qualitative and quantitative data collection will be analysed conform the requirements of a convergent parallel design. In a convergent parallel design, quantitative and qualitative data are concurrently collected in the same phase of the research process, and outcomes of both qualitative and quantitative analysis are combined during the overall interpretation [49].

## Discussion

The ACT-study aims to investigate the extent to which the geriatric care model has an effect on frail older adults’ quality of life. To our knowledge, it is one of the first European studies adopting a chronic care model approach to not only assess the effectiveness, but also the cost-effectiveness and the implementation process of an intervention for frail older adults living at home.

Carrying out the study in a real life primary care setting will provide insight into the generalizability of the geriatric care model, and may facilitate future implementation into routine practice. Moreover, the stepped-wedge design used in this study will allow us to introduce the intervention to all primary care practices participating in the study, so all frail older adults in the four allocation arms will eventually receive the geriatric care model. This has a number of advantages. First, the phased rolling out of the intervention will give us an opportunity to avoid having to withhold the geriatric care model from people who might benefit from it. Not only is this ethical argument compelling in itself, it has also played an important role in motivating primary care physicians to participate in the study. In addition, introducing the intervention to all four allocation arms offers methodological advantages. As mentioned in the effect evaluation paragraph, it will present us with the opportunity to take into account the effects of the duration of the intervention on outcome measures, Thus allowing us to differentiate between changes in outcomes due to time and due to the intervention.

In the years to come, the aging of the population will increasingly impose a strain on health care systems worldwide. It has become apparent that tackling emerging obstacles is essential in the securing of high-quality elderly care. By introducing the geriatric care model, we hope to contribute to the existing evidence on quality improvement and the effectiveness of integrated care models, and to present a solution for the many challenges facing care for older adults today.

## Abbreviations

ACT = Frail older adults: care in transition; ADL = Activities of daily living; ANOVA = Analysis of variance; CE = Cost-effectiveness; CU = Cost-utility; MICE = Multivariate imputation by chained equations; PRISMA = Program on research for integrating services for the maintenance of autonomy; RAI = Resident assessment instrument; QALY = Quality adjusted life years.

## Competing interests

The authors report no competing interests.

## Authors’ contributions

AJ, HvdH, HvH, and GN designed the initial study protocol and wrote the funding proposal. JT advised on the use of research methods. MM, EH and KvL worked out the initial study protocol and drafted the manuscript. MM wrote the background, the main part of the methods section and the discussion section. EH wrote the data collection and data analyses part of the effect evaluation section; KvL wrote the economic evaluation section. AJ supervised the writing process. All authors critically revised the manuscript for important intellectual content. All authors approved the final manuscript.
